# Predicting liver cancer on epigenomics data using machine learning

**DOI:** 10.3389/fbinf.2022.954529

**Published:** 2022-09-27

**Authors:** Vishalkumar Vekariya, Kalpdrum Passi, Chakresh Kumar Jain

**Affiliations:** ^1^ School of Engineering and Computer Science, Laurentian University, Sudbury, ON, Canada; ^2^ Department of Biotechnology, Jaypee Institute of Information Technology, Noida, India

**Keywords:** epigenomics, histone, DNA methylation, human genome, RNA

## Abstract

Epigenomics is the branch of biology concerned with the phenotype modifications that do not induce any change in the cell DNA sequence. Epigenetic modifications apply changes to the properties of DNA, which ultimately prevents such DNA actions from being executed. These alterations arise in the cancer cells, which is the only cause of cancer. The liver is the metabolic cleansing center of the human body and the only organ, which can regenerate itself, but liver cancer can stop the cleansing of the body. Machine learning techniques are used in this research to predict the gene expression of the liver cells for the liver hepatocellular carcinoma (LIHC), which is the third biggest reason of death by cancer and affects five hundred thousand people per year. The data for LIHC include four different types, namely, methylation, histone, the human genome, and RNA sequences. The data were accessed through open-source technologies in R programming languages for The Cancer Genome Atlas (TCGA). The proposed method considers 1,000 features across the four types of data. Nine different feature selection methods were used and eight different classification methods were compared to select the best model over 5-fold cross-validation and different training-to-test ratios. The best model was obtained for 140 features for ReliefF feature selection and XGBoost classification method with an AUC of 1.0 and an accuracy of 99.67% to predict the liver cancer.

## 1 Introduction

The pathogenesis of cancer stems from gene regulations responsible for unwanted cellular growth and division, which undergoes changes and mutations. Gene sets called oncogenes transform normal cell types into cancerous ones. Individual cancers have specific genetic anomalies ranging from epigenetic alterations, copy numbers (CNs), somatic mutations, and profiles. Disruption of gene expression (GE) can arise from a genotypic inheritance, cellular division, or environmental factors. GE changes lead to protein derangements that alter standard cellular behavior. Transformed cells begin abnormal proliferation, increasing in size to a tumor. Subsequently, certain tumors evolve to become cancers, and unique profile molecules identify individual types.

### 1.1 Identification of cancer

The advent of next-generation sequencing has paved the way for mapping out of entire genome data that single out variations and mutations specific to individual tumors. Copy number variations (CNVs) identify regions on genes that appear in different numbers in various individuals or separate cells in the same individual. A significant percentage of phenotypic variation is attributable to CNVs. Gene expression is altered by distorting code sequences, disrupting long-range regulations, or breaking gene dosages. Liver cancer growth is the second most regular reason for disease-related cancer around the world. It is one of the only neoplasms with a steadily increasing incidence and mortality (Llovet et al., 2009). Liver cancer comprises a heterogeneous group, with an unfavorable prognosis that ranges from hepatocellular carcinoma (HCC) and intrahepatic cholangiocarcinoma (iCCA) to mixed hepatocellular cholangiocarcinoma (HCC-CCA), fibrolamellar HCC (FLC), and the pediatric neoplasm hepatoblastoma (Torre et al., 2015).

### 1.2 Liver hepatocellular carcinoma

Liver hepatocellular carcinoma is touted as the most aggressive form of liver cancer with etiologies ranging from chronic infections (HBV and HCV), diabetes, and metabolic diseases to autoimmune hepatitis (Mavilia and Wu, 2018). Genomic profiling studies show deviation of DNA. CNs play a pivotal regulatory role in the progression, and the subsequent transcriptional deregulation is a likely driver in advance. Chemotherapy, radiotherapy, and surgeries have only been able to limit the tumor mass. Further relapse has been seen after the completion of therapy. The last decade has seen a significant increase in the execution of cancer stem cells that have led to significant progression in hepatocellular carcinoma. Cancer stem cells are found in many hematological and solid human tumors. The dual role of embryonic stem cell development and tumor suppression, significant surface markers, and pathways to modulate stem cells were found in a recent study (Mishra et al., 2009). Cancer cell carcinoma studies have shown that a population apart from self-renewing/differentiating capacity also increases resistance to radiation and chemotherapy. They are the leading cause of tumor relapse. Liver cancer stem cells (CSCs) are considered a master regulator of hepatocellular carcinoma initiation and hepatic progenitor cells could form the basis of LIHC.

### 1.3 Gene expression and research

The latest computation techniques and novel bioinformatics have been continuously used in new oncogenic research studies, and high-throughput technologies (microarray analysis and RNA sequencing) are playing a major role in identifying novel tumor markers by the researchers for cancer diagnostics and targeted treatment. Yang et al. (2017) studied (using data from 50 paired samples on other bioinformatics platforms) dysregulated genes and signaling pathways in LIHC. Machine learning (ML), deep learning (DL), and natural language processing (NLP) are used to accelerate these types of research and studies. NLP uses the interaction between computers and natural language to gather sporadic laboratory and clinical data to speed up the scientific clinical practice. Machine learning and deep learning methods can build accurate models that can predict the diseases in the patients on the processed features from gene expressions and various data based on methylation, histone, RNA sequences, etc. LIHC refers to the malignant tumor of liver cells. It accounts for 85% of all hepatic cancers (Sherman, 2019). Epidemiological analysis indicates that the high fatality rate is due to late diagnosis and poor prognosis. In the United States, liver cancer is increasing briskly among both men and women. From 2006 to 2015, a steep increase of 3% is observed every year in HCC incidences. The American Cancer Society (ACS) had predicted that in 2020, there will be 42,810 new incidents of liver cancer, among which almost three-fourths will be of HCC (Cicalese, 2020). People with chronic hepatitis B, hepatitis C, alcohol addiction, or who have been exposed to toxins such as aflatoxin, are usually suspected of HCC (Yang et al., 2019).

Distinct epigenetic signatures catalyzed by various epigenetic modifiers control developmental regulation of gene expression. Epigenetic control of chronic liver diseases advances our understanding of critical roles of DNA methylation/demethylation, histone acetylation/deacetylation, histone phosphorylation and mRNA, and micro RNA involved in these alternative pathways.

### 1.4 Wnt signaling pathway

In tumor development and differentiation, the Wnt signaling pathway exhibits a vital role. Accumulation of β-catenin in HCC patients indicates the activation of the Wnt signaling pathway. Almost 62% of HCC patients displayed an excessive collection of β-catenin (Inagawa et al., 2002). β-Catenin mutations also demonstrate the enhancement of HCC. Due to the disruption of the adenoma polyposis coli (APC) gene in mice, the Wnt β-catenin pathway was activated, which led to the development of HCC (Colnot et al., 2004).

## 2 Materials and methods

To represent the cycle of information investigation of a cellular breakdown in the liver, we constructed a stream scorch to decipher the means of this investigation. Four distinct kinds of datasets of epigenomics data incorporate CpG methylation data, histone modification data, human genome data, and RNA-Seq data are utilized for information examination. The datasets were downloaded from the RTCGA library, and a few preprocessing steps were performed by R and necktie programming (Kosinski and Biecek, 2022). By using the record ID of each dataset, all the datasets were joined and a model was created utilizing R. The data were pre-processed to reduce the dimensionality and extract the most relevant features by applying nine feature selection methods, namely, principal component analysis (PCA), correlation-based feature selection (CFS), chi-squared, MI-score (mutual information), recursive feature elimination (RFE), SelectK, embedded method with logistic regression (EMLR), embedded method with random forest (EMRF), and ReliefF. The data were split into different ratios for training and testing with 90:10, 80:20, 70:30, and 60:40. The data were validated using 5-fold cross-validation to avoid overfitting. Additionally, statistical analysis was used to ensure that the results were distinct and not obtained by chance. Eight unique classifiers were applied, namely, radial SVM, linear SVM, XGBoost, naïve Bayes, random forest, K-nearest neighbor (KNN), multilayer perceptron (MLP), and decision tree.

Data processing is performed by developing custom R Script whereas classification, as well as feature selection, is performed using data mining software—Weka (Frank et al., 2016).

### 2.1 Dataset selection

We used the R and open-source library RTCGA to download all the necessary datasets from The Cancer Genome Atlas (TCGA) Data Portal. TCGA data portal provides a platform for academic researchers and scientists to search, download, and analyze datasets generated by TCGA. It contains clinical information, genomic characterization data, and high-level sequence analysis of the tumor genomes from DNA methylation, RNA, and mRNA sequences to histone modification data with epigenomics, human genome data, and clinical information of the patients for the integration of all the data. The key is to understand genomics to improve cancer care. RTCGA is an open-source R package which is available and provided through Bioconductor. It also works as an interface for the integrative analysis of RNA-seq or microarray-based gene transcription and histone modification data obtained by ChIP-seq. The package provides methods for data preprocessing. Other packages were also used, such as TCGAbiolonks and SummarizedExperiments, ShortRead, Rsubread, BSgenome.Hsapiens. UCSC.hg19, EnsDb.Hsapiens.v75, and DESeq2 (Colaprico et al., 2015) (Morgan et al., 2022) (Morgan et al., 2009) (Liao et al., 2019) (Team, 2020) (Rainer, 2017) (Love et al., 2014). The RTCGA package, apart from an interface to the TCGA in R, allows researchers to transform TCGS data into a form that is convenient to use in the R statistical package.

#### 2.1.1 DNA methylation data

From TCGA, methylation data from Illumina’s Infinium HumanMethylation450 Bead Chip (Illumina 450 k) were obtained. The addition of a methyl group to the fifth position of genomic cytosine forms 5-methylcytosine (5 mC), often called the fifth base and is a widely studied epigenetic mark. In mammals, 5 mC predominantly occurs in the CpG context. Still, in other organisms, it can occur in CHG and CHH contexts where H is an A, C, or T. Also, 5 mC is prevalent throughout various tissues with 60%–80% of CpGs being methylated. CPG’s genomic instructions and coding of exons were acquired from the code provided by Li et al. (2015). We re-noted the protein-coding properties using the exons, coding DNA structures (CDS), and gradually deleted the exhaustive data from different transcript areas. The transcript areas include all introns (with uncommon first and last intron classes), only non-translated positions in the 5′ and 3′ headings (5′ UTR and 3′ UTR, individually), as first and last exons and a “single exon” or “single intron” designation for transcripts which only had one exon or one-half exon A intron.

#### 2.1.2 Histone data

HpeG2 cell line was considered for three schemes of histone ChIP-seq data, which were H3k4me3, H3k27me3, and H3k36me3 from hepatocellular carcinoma tissue (0.2% EtOH therapy). The data were chosen from the UCSC genome browser, which is a joint effort of the ENCODE project through the UCSC Chrome genome to http:/genome.ucsc.edu. Data from the large were downloaded from Raw ChIP-seq. The data were processed using R efficiently to ensure the accuracy of all system standardization using a custom R script. After the arrangement of crude histone marker information, the adjusted histone marker peruses were converged with the portions of every record utilizing the multicov work from the BEDTools bundle (Quinlan and Hall, 2010). The histone peruses were then standardized per 1,000 bp length of each fragment per 1 million adjusted read library. Similar to the CpG methylation, the histone marker adjustment highlights were extricated on a fragment by-section basic. The numbers addressing particular histone H3 methylation markers are four for H3k4me3, 27 for H3k27me3, and 36 for H3k36me3. Accordingly, highlights are named as a segment cell type and histone change type (for example, first_exon_A4). To analyze histone adjustment between the disease and non-malignant growth cell types, the distinctions of the peruses between them were isolated by the normal of the two (for example, an element named first_exon_A4_minus_S4_divavg).

#### 2.1.3 Human genome data

We extracted the nucleotide composition data from the hg19 genome through Bioconductor’s BSgenome.Hsapiens.UCSC.hg19 open-source library (Team, 2020), which was initially decided and selected from the study of the UCSC genome browser, which is produced by clinicians having three classes of species: vertebrates, primates, and placental. A custom R script was used to process the data using Rsubreads and other open-source packages (Liao et al., 2019).

#### 2.1.4 RNA-seq data

RNA-Seq gene expression data from liver cancer samples with coupled CpG methylation data were already downloaded from TCGA Research Network using RTCGA for hepatocellular carcinoma. The data are selected from TCGA, which is produced by clinicians. Differential expression analysis was performed with the DESeq2 package in R (Love et al., 2014). The expression of a gene was taken as binary outcomes: either up-regulated or down-regulated, once it passed two thresholds: 1) having an adjusted *p*-value < 0.05, FDR cut off of 0.01 after having an absolute value of log2 fold change or logFC <0.5. As a result, 6,794 genes were selected as “differentially expressed” genes.

### 2.2 Feature extraction

In the last decade, feature selection has received a tremendous amount of attention from machine learning researchers. Feature selection or feature engineering aims at finding the best subset of features from the total number of features that can represent the input data efficiently and can still provide good prediction results for machine learning modelling. Feature selection uses a search algorithm to find one or more informative subsets of features that indicate to predefined criteria. Feature selection should be able to extract the important or relevant features (i.e., the features relevant to the given prediction task). If a problem has N features, the number of all subsets of features is equal to 2 N. Therefore, the optimal set of features is one (or could be more) of an exponential number of possible subsets and comparing all of these subsets will locate the best intractable for N > 20 (Colaprico et al., 2015). Feature selection determines the feature relevance according to an evaluation criterion associated with the given method. In general, feature selection methods can be divided into three types: filter methods, wrapper methods, and embedded methods.

(a) Filter methods involve the methods that perform feature selection before building the classifier and do not incorporate learning.
(b) Wrapper methods incorporate machine learning in measuring the quality of the subsets of features without incorporating knowledge about the particular structure of the classification or regression function.
(c) Embedded methods are different from filter and wrapper methods in that the learning part and the feature selection part cannot be separated in the embedded methods.

**FIGURE 1:**
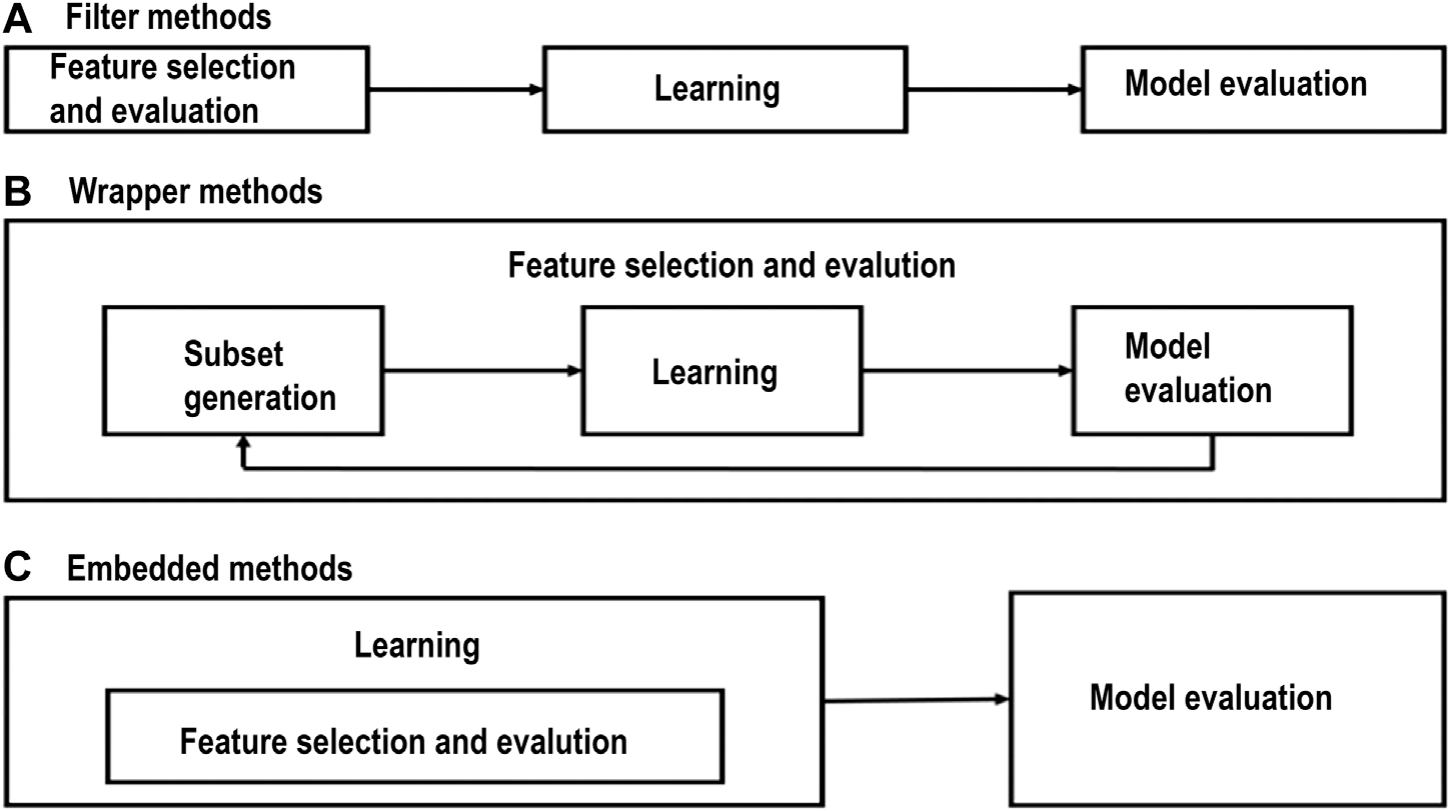
**(A)** Filter methods, **(B)** wrapper methods, and **(C)** embedded methods (Naqvi, 2011; Morgan et al., 2022).

Figure 1 shows the three categories of feature extraction methods.

#### 2.2.1 CpG methylation features

Differential articulation of the methylated CpG locales was prepared utilizing the limma library in R (Smyth, 2005). In particular, the work top table was utilized to decide the log overlap change (logFC) between the malignant growth and typical tissues as well as the normal methylation (avgMval) of each CpG site over the two kinds of tissues. A positive logFC demonstrates hypermethylation and a negative logFC demonstrates hypomethylation. Extra section-based highlights were additionally considered. These incorporate the number of hypermethylated (numHyper) and hypomethylated tests (numHypo) on a section of a given record. For instance, first_exon_numHyper alludes to the number of hypermethylated tests on the principal exon. Two other kinds of highlights are normal of logFC and avgMval of all CpG tests on a fragment of the record, for example, the normal logFC of all tests on the primary exon of the guaranteed record (first_exon_avglogFC).

Extraordinary exertion was paid to process separations of CpG tests to exon-intersections. Given that at least one CpG destination may exist on the individual exon fragments of a record (counting the first and last exons), transcript-level most extreme, least and normal separations of any hyper/hypo-methylated test to the closest 5′ or 3′ exon-exon intersection were processed (maxHypoTo5, min- HypoTo5, avgHypoTo5, maxHypoTo3, minHypoTo3, avgHypoTo3, maxHyperTo5, minHyperTo5, avgHyperTo5, maxHyperTo3, minHyperTo3, and avgHyperTo3).

#### 2.2.2 Histone marker change feature

The histone peruses were then standardized per 1,000 bp length of each fragment per 1 million adjusted read library. Similar to the CpG methylation, the histone marker adjustment highlights were extricated on a fragment by-section basic. The numbers addressing particular histone H3 methylation markers are four for H3k4me3, 27 for H3k27me3, and 36 for H3k36me3. Accordingly, highlights are named as a segment cell type and histone change type (for example, first_exon_A4). To analyze histone adjustment between the disease and non-malignant growth cell types, the distinctions of the peruses between them were isolated by the normal of the two (for example, an element named first_exon_A4_minus_S4_divavg).

### 2.3 Feature selection

In the last decade, feature selection has received a tremendous amount of attention from machine learning researchers. Feature selection or feature engineering aims at finding the best subset of features from the total number of features that can represent the input data efficiently and can still provide good prediction results for machine learning modeling. Feature selection uses a search algorithm to find one or more informative subsets of features that indicate to predefined criteria. One significant issue while applying a massive epigenetic dataset onto the unmistakable classifier is excess features.

A couple of sorts of examinations exhibited that the use of a component choice procedure can somehow improve the precision and besides decline the excess. In this research, feature selection methods were applied to reduce the features and include the following: PCA (principal component analysis), CFS (correlation-based feature selection), chi-squared, ReliefF, MI score (mutual information), RFE (recursive feature elimination), EMLR (embedded method with logistic regression), EMRF (embedded method with random forest), and SelectK.

### 2.3.1 Chi-squared

Chi-squared χ2 is a filter method that evaluates features individually by measuring their chi-squared statistic with respect to the classes. In statistics, the χ2 test is applied to test the independence of two events, where two events A and B are defined to be independent if (AB) = (A) (B) or, equivalently, P (A|B) = P(A) and P(B|A) = P(B). In feature selection, the two events are the occurrence of the term and circumstance of the class. We then rank terms with respect to the following quantity:

$$x^{2}=\sum \frac{{\left(O_{i}-E_{i}\right)}^{2}}{E_{i}},$$ (1)

where O is the observed value, and E is the expected value. χ2 is a measure of how much expected counts E and observed counts O deviate from each other. A high value of χ2 indicates that the hypothesis of independence, which implies that expected and observed counts are similar is incorrect (Liao et al., 2019).

#### 2.3.2 Mutual information

Mutual information (MI) is another filter-based method that measures the amount of information that one random variable has about another variable. MI between two random variables is a non-negative value, which measures the dependency between the variables. It is equal to zero if and only if two random variables are independent, and higher values mean a strong dependency/association. Mutual information “is not concerned” with whether the univariate association is linear or not.

#### 2.3.3 Recursive feature elimination

Recursive feature elimination (RFE), a wrapper method, is used to select features by recursively considering smaller and smaller sets of features based on an external estimator that assigns weights to features (i.e., the coefficients of a linear model). First, the estimator is trained on the initial set of features, and the importance of each feature is obtained either through a coefficient attribute or through feature importance attribute. Then, the least important features are pruned from the current set of features. That procedure is recursively repeated on the pruned set until the desired number of features to select is eventually reached (Team, 2020).

#### 2.3.4 Embedded methods–variable importance-based methods

Algorithms such as logistics regression-based SelectKBest features or decision trees and random forest tree-based algorithms can also be used to extract the features from high dimensional features. The methods are usually based on the following approaches. Permutation-based variable importance: this algorithm consists of permuting the variables at test time and looking at the accuracy loss. This technique is part of the wrapper algorithms. It can thus be used with any learning algorithm.

#### 2.3.5 Dimensionality reduction with principal component analysis

Dimensionality reduction is the process of reducing the number of features used in machine learning algorithms. This can be used to increase the accuracy and the performance of machine learning algorithms. One form is to perform data compression. For example, transform 3D data into 2D data and eliminate a feature or dimension. It can also be used to reduce dimensions to be able to visualize data efficiently. Dimensionality reduction can be used to speed up the time it takes for other learning algorithms to learn. By using dimensionality reduction, the number of features or the amount of training samples is reduced, which reduces the running time of the training, but the compressed data still retain the same information as the uncompressed data. The algorithm is formulated as a minimalization problem. When given N-dimensional data and N-1 dimensional data are preferred, the algorithm tries to find the correct N-1 dimensional value so that the projection is the closest to the original data. Before this algorithm is run, the features of the data should be scaled so that all features are on a similar scale. This can be carried out by using mean normalization. In order to reduce the dimension from n to k, the covariance matrix should be computed. From this matrix, the eigenvectors need to be computed using singular value decomposition. From these values, only the first k values are going to be used and be multiplied with the training data.

### 2.4 Classifiers

After applying feature selection for dimensionality reduction, classification algorithms are applied to quantify and look at the qualification between different feature selections. Any grouping methodology uses a ton of parameters to portray each object. These highlights are noteworthy to the information being inspected. Classification is a supervised learning technique where labels are present on the information and the classification model predicts the gene expression of the dataset. In this research, eight classifiers were applied and their performance was evaluated, XGBoost (XG), random forest (RF), naïve Bayes (NB), K-nearest neighbor (KNN), multilayer perceptron (MLP), Decision tree (DT), and radial and linear support vector machines (SVMs).

#### 2.4.1 XGBoost

XGBoost is a choice tree-based gathering machine learning calculation that utilizes a slope boosting system. In expectation issues including unstructured information (pictures, text, and so on), artificial neural organizations will, in general, beat any remaining calculations or systems. In any case, with regards to little-to-medium organized/plain information, decision tree-based calculations are viewed as top tier at this moment.

### 2.4.2 Random forest

Random forests are utilized for grouping and relapse by developing decision trees during preparations. It in this way settles on the choice tree dependent on the subsets that are chosen randomly from the dataset.

### 2.4.3 Naïve Bayes

Naïve Bayes classifiers are the probabilistic classifiers that work on Bayes’ theorem principle. The algorithm has independent assumptions among the features. On an abstract level, given a vector representing some features, the naive Bayes will assign a probability to all possible outcomes (also called classes) given this vector. It is a conditional probability model. Based on Bayes’ theorem, a reliable and computable model can be constructed for all the possibilities that need to be generated. From the naive Bayes probability model, we can then construct a classifier. The naive Bayes classifier usually combines the probability model with a decision rule. This rule will define which hypothesis is to be picked. A common rule is simply picking the one with the highest probability, which is also called the “maximum posterior” (MAP) decision rule (Liao et al., 2019) (Rainer, 2017).

### 2.4.4 K-nearest neighbor

It is a non-parametric model of classification, which can, likewise, be utilized for regression. It stores all the potential cases and uses them to order the new cases dependent on the comparability record. The K-nearest neighbors or the KNN algorithm is a supervised learning algorithm that computes the classification by looking at the classes of the training data’s K-nearest neighbors. When training the algorithm, the input data and classes are stored. There are multiple methods to compute the distance between data. Euclidean distance can be used for continuous data. For discrete variables, another metric can be used, such as the Hamming distance. There are many different methods for computing the distance. A significant drawback of the KNN algorithm is the weakness of skewed data. Skewed data are data that belong to a class that is underrepresented in the complete dataset. Since the class is chosen based on the most popular nearest class, these popular classes may dominate the prediction. A straightforward solution is to gather more data. The problem can be overcome more realistically by taking the distance between the input data and the neighbors into account. If this does not work, it is possible to use learning algorithms to select the best data from the dataset so that no class is underrepresented. These methods are far more advanced and have a higher computational cost.

### 2.4.5 Multilayer perceptron

A multilayer perceptron (MLP) is a feedforward artificial neural organization that creates a bunch of yields from a bunch of data sources. An MLP is described by a few layers of information hubs associated with a coordinated diagram between the information and yield layers. MLP utilizes backpropagation for preparing the organization. MLP is a deep learning method. Artificial neural networks work on a simplified model of how human brains work. They generate layers of nodes, each having certain input and output values. An activation function triggers the nodes (also called neurons). This function can take many shapes and be triggered by a combination or a series of inputs of a neuron. Once the activation function has been triggered, the neuron will send its output signal throughout the outgoing channels.

### 2.4.6 Decision tree

A decision tree is a simple representation for classifying examples. It is supervised machine learning where the information is continuously part as per a specific parameter. A decision tree consists of nodes: test for the estimation of a specific trait. Edges/branch: correspond to the result of a test and interface with the following hub or leaf. Leaf hubs: terminal hubs that foresee the result (speak to class names or class circulation).

### 2.4.7 Radial and linear SVMs

Linear SVM is a quick data mining calculation for managing multiclass classification issues out of an incredibly enormous arrangement of data. The arrangement of numerical capacities called kernel is applied to achieve the cycle. Normally support vector machines, as well as general logistic regression work with linear classifiers. A classifier can be linear or non-linear. A linear classifier has a decision boundary, which is a linear function. This can be seen by visualizing that there is a straight line drawn through the data. On one side of this line, the data belong to one class and the data on the other side of this line belongs to another class (Love et al., 2014).

### 2.5 Cross-validation

Cross-validation is a statistical method of evaluating and comparing learning algorithms by repeatedly partitioning the given dataset into two disjoint subsets: the training and the test subsets. The training subset is used to build the classifier model, and then the samples belonging to the test subset are used to test the trained model. The process is repeated with several partitions and gives an estimate of the classification performance. The most common form of cross-validation is k-fold cross-validation.

K-fold cross-validation: the k-fold cross-validation partitions the given dataset into k equally sized subsets. Then, training is performed on k−1 subsets and testing is performed on the remaining subset. This process is repeated k times (folds) with each subset taken as a test set in turn (Morgan et al., 2009).

Leave-one-out cross-validation: in this method, we use k-fold cross-validation, where k is equal to the number of samples in the dataset. In each “fold,” n−1 samples are used as a training set, and a single sample is used for testing. This procedure is repeated for all samples. This method is computationally expensive as it requires the construction of n different classifiers. However, it is more suitable for smaller datasets (Morgan et al., 2009).

## 3 Results

The training and testing of the models were conducted on various assumptions. There were total nine methods of the feature selection. The models were trained on 5-fold cross-validation as there are lesser observations in the data, the 5-fold cross-validation will allow the models to train on all data as well as testing on all data utilizing the all datasets and providing us the average results for each fold. The models were trained with 5-fold cross-validation to ensure overfitting does not happen. The evaluation metrics used to evaluate the performance of the classifiers include accuracy, sensitivity, specificity, and area under receiver operating characteristics (AUC). We considered the accuracy score, confusion matrix, sensitivity score, and specificity score as the evaluation metrics for all the machine learning models. The percentage ratio for feature selection is raw, 95%, 90%, 85%, 80%, 75%, 70%, 65%, 60%, 55%, and 50%. Furthermore, the results are concluded using eight different classifiers. Also, 5-Fold cross validations are performed for different training and testing ratios (90:10, 80:20, 70:30, and 60:40). The epigenomics data were compiled using four sources, namely, methylation data, histone H3 marker ChIP-seq data, human genome data, and RNA-Seq gene expression data. In total, 1,000 features were calculated. Nine feature selection methods from the three categories of feature selection algorithms (filter, wrapper, and embedded methods) were applied.

**FIGURE 2:**
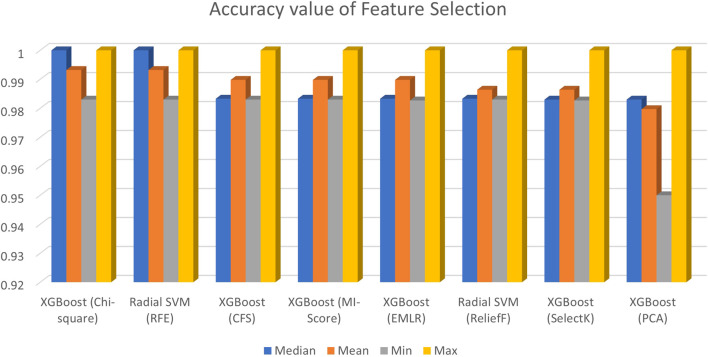
Accuracy value of feature selection.

As it can be seen from Figure 2, the accuracy results of all feature selection methods, six out of eight models perform well across the various machine learning algorithms. KNN, SVM linear, and MLP have higher error rates in many cases. XGBoost, random forest, decision tree, naïve Bayes, and SVM radial are found to be the best models across the various feature selection methods. In order to look into the accuracy scores in more detail and sort the models and feature selection methods based on mean accuracy across the various folds, custom python scripts were written for reading the saved outputs which were stored in different sub-directories and a table was created containing the model, feature selection method, median accuracy, mean accuracy, and minimum and maximum accuracy which is shown in Figure 2.

Table 1 shows the highest accuracy and AUC values for the eight classifiers. It is evident that the ReliefF feature selection gives the best performance with 100% AUC, whereas XGBoost is selected to be the best classifier with 140 features. After trying out different models, the accuracy was obtained by median, mean, minimum, and maximum. EMLR also gives the best AUC of 100% and 0.9899 accuracy with 140 features with the XGBoost classifier. Table 2 shows the list of best features that help to obtain best AUC and Accuracy.

**TABLE 1:** Summary table for best performance of each selection method.

Feature selection	Classifier	AUC	Accuracy	Number of features
ReliefF	XGBoost	1	0.9967	140
CFS	XGBoost	0.9974	0.9854	110
EMLR	XGBoost	1	0.9899	140
MI-score	XGBoost	1	0.9893	140
Chi-squared	XGBoost	1	0.9892	140
RFS	RSVM	1	0.9892	140
EMRF	Random forest	1	0.9891	140
PCA	RSVM	0.9961	0.9873	140
SelectK	XGBoost	1	0.9871	140

**TABLE 2:** List of best features that help to obtain best AUC and accuracy.

1TSS1500	24
UTR5	11
TSS200	19
UTR3	09
CDS	10
FIRST EXON	12
LAST EXON	07
FULL TRANSCRIPT	15
HYPERMETHYLATED	16
SINGLE EXON	09
SINGLE INTRON	08
**TOTAL**	**140**

Bold values shows the total number of features.

**FIGURE 3:**
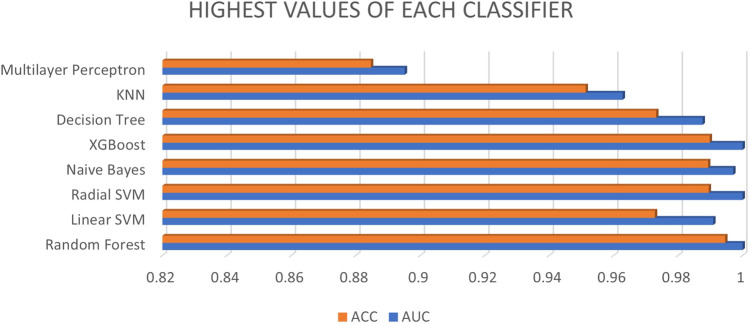
Highest values of each classifier.

As per aforementioned Figure 3, the database with different ratios and feature ratios as well as XGBoost is selected as the best classifier, along with random forest and radial SVM, while multilayer perceptron has low AUC and accuracy compared to other classifiers.

**FIGURE 4:**
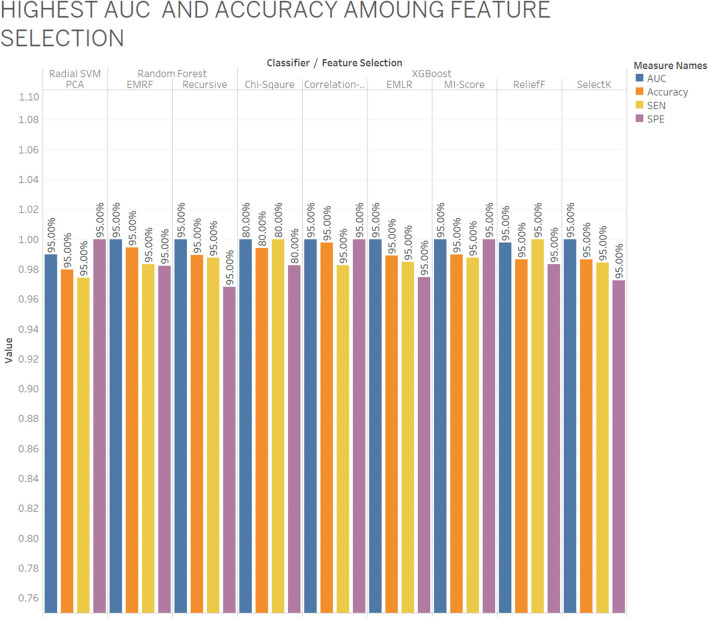
Highest AUC and accuracy among feature selection.

As per aforementioned Figure 4, the XGBoost classifier is selected as a best classifier with chi-squared, CFS, EMLR, MI-score, ReliefF, SelectK feature selection and EMRF and RFS with the random forest classifier also give the best AUC and accuracy with specificity and sensitivity where blue color indicates AUC, orange color as accuracy, yellow color as sensitivity, and purple color as specificity. Among nine out eight give best AUC with almost 95% and 80% ratio. All four types of data (CpG methylation, histone modification, human genome, and RNA-seq data) included 1,000 features (558 DNA methylation data, 207 histone modification data, and 235 nucleotide composition) from 14,899 features. They all provide relevant information that contributes to better prediction of the gene expression.

Overviewing the best number of features with feature selection and classifiers, which were obtained with AUC and accuracy.

### 3.1 Grid-search based hyper-parameter tuning of the models

The next step with model building and evaluation was to use grid-search-based hyper-parameter tuning for finding the best combination of parameters to find the best model. For this approach, a custom R script was written. The training was conducted using 80% data and 20% data were used for testing of the model. SMOTE oversampling was also used in this approach, which was also used with the model comparison strategy. SMOTE balances the data and the imbalanced classes. The best hyper-parameter corresponds to the best model of same kind with a number of different values supplied for the respective model.

### 3.1.1 Correlation-based feature selection

The following best parameters were found for different models for the CFS method.

• KNN: (K = 9)
• XGBoost: (Alpha = 1e-04, lambda = 50)
• MLP: (*n* = 10)
• Naive Bayes: (use_kernel = False)
• Random forest: (m_try = 10, 200)
• Decision tree: (max depth = 1)
• SVM linear: (L2 and C = 1)
• SVM radial: (sigma = 1)

#### 3.1.2 Chi-squared-based feature selection

The following best parameters were found for different models for the chi-squared method.

• KNN: (K = 7)
• XGBoost: (Alpha = 1e-02, lambda = 100)
• MLP: (n = 7)
• Naive Bayes: (use_kernel = True/False)
• Random forest: (m_try = 200)
• Decision tree: (max depth = 1/2)
• SVM linear: (L and C = 200)
• SVM radial: (sigma = 1/2/3/4)

#### 3.1.3 Mutual information-based feature selection

The following best parameters were found for different models for the CFS method.

• KNN: (K = 9)
• XGBoost: (Alpha = 1e-04, lambda = 50)
• MLP: (*n* = 1)
• Naive Bayes: (use_kernel = False)
• Random forest: (m_try = 200)
• Decision tree: (max depth = 1)
• SVM linear: (L2 and C = 2)
• SVM radial: (sigma = 0.5)

#### 3.1.4 Principal component analysis

The following best parameters were found for the different models for the PCA method.

• KNN: (K = 5)
• XGBoost: (Alpha = 1e-04, lambda = 50)
• MLP: (n = 10)
• Naive Bayes: (use_kernel = False)
• Random forest: (m_try = 40)
•Decision tree: (max depth = 3)
• SVM linear: (L1 and C = 0)
• SVM radial: (sigma = 0.5/1)

#### 3.1.5 ReliefF

The following best parameters were found for different models for the Relief method.

• KNN: (K = 4)
• XGBoost: (Alpha = 1e-02, lambda = 100)
• MLP: (n = 7)
• Naive Bayes: (use_kernel = False)
• Random forest: (m_try = 200)
• Decision tree: (max depth = 1/2)
• SVM linear: (L1 and C = 1)
• SVM radial: (sigma = 1)

#### 3.1.6 Recursive feature elimination

The following best parameters were found for different models for the RFE method.

• KNN: (K = 5)
• XGBoost: (Alpha = 1e-02, lambda = 100)
• MLP: (*n* = 7)
• Naive Bayes: (use_kernel = False)
• Random forest: (m_try = 0)
• Decision tree: (max depth = 1/2)
• SVM linear: (L1 and C = 2)
• SVM radial: (sigma = 0)

#### 3.1.7 Embedded method with logistic regression

The following best parameters were found for different models for the EMLR method.

• KNN: (K = 5)
• XGBoost: (Alpha = 1e-02, lambda = 100)
• MLP: (n = 7)
• Naive Bayes: (use_kernel = False)
• Random forest: (m_try = 100)
• Decision tree: (max depth = 1/2)
• SVM linear: (L1 and C = 4)
• SVM radial: (sigma = 0)

#### 3.1.8 Embedded method with random forest

The following best parameters were found for different models for the EMRF method.

• KNN: (K = 5)
• XGBoost: (Alpha = 1e-02, lambda = 100)
• MLP: (n = 1)
• Naive Bayes: (use_kernel = False)
• Random forest: (m_try = 0)
• Decision tree: (max depth = 1/2)
• SVM linear: (L1 and C = 2)
• SVM radial: (sigma = 1)

#### 3.1.9 Embedded method with SelectKBest

The following best parameters were found for different models for the SelectK method.

• KNN: (K = 8)
• XGBoost: (Alpha = 1e-02, lambda = 100)
• MLP: (*n* = 8)
• Naive Bayes: (use_kernel = False)
• Random forest: (m_try = 200)
• Decision tree: (max depth = 1,2)
• SVM linear: (L2 and C = 1)
• SVM radial: (sigma = 4)

### 3.2 Statistical analysis

Machine learning models are statistical and probabilistic which work on the basis of calculations and statistical methods and identifying patterns in the data to make predictions. There are chances that observations which consist of drawing samples from a population indicates an effect which can occur because of sampling errors. If the observed effect indicates a *p*-value < 0.05 (95% confidence interval (CI)), a conclusion can be made out of the assumptions that the observed effect reflects the characteristics of the entire population, on the other hand, if the *p*-value is greater than 0.05, the observed effect does not reflects the characteristics of the entire population.

However, to perform this analysis, the data should satisfy the following assumptions: 1) normal distribution, 2) homogenous variance, 3) absence of significant outliers, and 4) independence of observations (Li et al., 2015). Shapiro–Wilk normality analysis (Quinlan and Hall, 2010) was performed to investigate for data normality and Levene’s analysis (Frank et al., 2016; Kim and Cribbie, 2018) to check for homogeneous variances. The alternate hypothesis (H1) is accepted and H0 is rejected if a statistically significant performance difference (*p* < 0.05) is found to exist. One-way ANOVA is an omnibus test and needs a *post hoc* study to identify the specific ensemble methods demonstrating these statistically significant performance differences. In this study, a Tukey *post hoc* test (Quinlan and Hall, 2010) was also performed to identify the models demonstrating these statistically significant performance differences. An R custom script was developed to perform statistical analyses.

**FIGURE 5:**
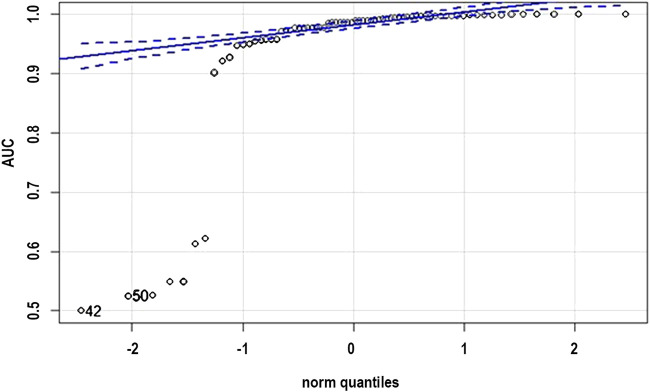
QQ plot normality check for the distribution.

The normality check was conducted on the AUC scores represented in the Table 1. It cannot be said that “the sample does not have a normal distribution,” but only “we can reject the hypothesis that the sample comes from a population which does not have a normal distribution.” But the sample does not have a fair normal distribution looking at the qqplot as shown in Figure 5, but we would not expect it to, as it is only a sample.

The results below show the output of the Bartlett’s test.

Bartlett test of homogeneity of variances.

Data: AUC by model.

Bartlett’s K-squared = 207.82, df = 7, *p*-value < 2.2e-16.

From the output we can see that the *p*-value is less than the significance level of 0.05. This means we can reject the null hypothesis that the variance is the same for all models. This means that there is good evidence to suggest that the variance in AUC is different for the different models. The following results show the output for the Shapiro’s test.

Shapiro–Wilk normality test.

Data: df$AUC.

W = 0.46391, *p*-value = 8.91e-15.

From the output, the *p*-value which is less than 0.05 implying that the distribution of the data is quite different from normal distribution. We also performed the Levene’s test. In this test again, the null hypothesis H0 is that all variances are equal.

The following output shows the one-way ANOVA for models and feature selection methods. One-way ANOVA for models and AUC is shown in the results below.

Df Sum Sq Mean Sq F value Pr (>F).

Model 7 0.9147 0.13067 29.56 <2e-16 ***.

Residuals 64 0.2829 0.00442.

---

Signif. codes: 0 “***” 0.001 “**” 0.01 “*” 0.05 “.” 0.1 “ ” 1

The *p*-value is lower than the usual threshold of 0.05. So, we are confident to say that there is a statistical difference between the models. One-way ANOVA for the feature selection method and AUC is shown in the results below.

Df Sum Sq Mean Sq F value Pr (>F)

Feature.Selection.Method 8 0.0348 0.004344 0.235 0.983.

Residuals 63 1.1628 0.018457.

The *p*-value is greater than the usual threshold of 0.05. So, we are confident to say that there are no statistical differences between the feature selection methods.

## 4 Conclusion and future work

In this research, a methodology was proposed to predict liver cancer using epigenomics data, feature extraction methods, and machine learning methods. Using the Illumina Infinium HumanMethylation450 K Bead chip CpG methylation range, this technique utilizes information from combined pulmonary disease and neighboring ordinary organs in the Cancer Genome Atlas (TCGA) and histone alteration marker ChIP-seq from the ENCODE initiative. It sees a comprehensive list of characteristics covering the four classes of CpG methylation, histone H3 methylation alteration, human genome, and RNA-Seq data. Different techniques and choices of features selection methods (chi-squared, CFS, MI-score, RFE, EMLR, ReliefF, SelectK, PCA, and EMRF) and eight different classifiers (XGBoost, radial SVM, naïve Bayes, random forest, decision tree, linear SVM, KNN, and MLP) were implemented, and classification was contrasted in the training-to-testing ratios and cross-validation methods to select the best model. The results show that a selection of 140 features gave the best model. XGBoost with ReliefF feature selection methods performed as the best model with 100% of AUC and 99.67% of accuracy toward the correct prediction of liver cancer.

As a future work, other cancer cell diagnoses such as breast cancer, bone cancer, blood cancer, and oral cancer can be used for predicting the gene expression. The accuracy level and methodology of this research and analysis results can help to work more on such datasets. More profound choices can be made on merging the datasets, using all histone modifications, and collecting and building capacity to harness more data for more patients and develop a predictive model that is highly accurate as this model and can be used in real-time predictions. A predictive model can be built with multiple types of epigenomics data obtained from the same samples in the ideal setting.

## Data Availability

The original contributions presented in the study are included in the article/Supplementary Materials; further inquiries can be directed to the corresponding author.
